# A disproportionality analysis of FDA adverse event reporting system events for misoprostol

**DOI:** 10.1038/s41598-025-86422-z

**Published:** 2025-01-19

**Authors:** Li Yang, Wenting Xu

**Affiliations:** 1 Department of Obstetrics and Gynaecology, Zhangjiagang TCM Hospital Affiliated to Nanjing University of Chinese Medicine, Zhangjiagang, Jiangsu China; 2 Department of Reproduction, Zhangjiagang TCM Hospital Affiliated to Nanjing University of Chinese Medicine, Zhangjiagang, Jiangsu China

**Keywords:** FAERS, Adverse event, Pharmacovigilance, Misoprostol, Disproportionality analysis, Drug regulation, Drug safety, Pharmacology, Drug regulation

## Abstract

Misoprostol was originally used to treat gastric ulcers, and has been widely used in abortion, cervical maturation, induced labour and postpartum hemorrhage. But there are still many undetected adverse events (AEs). The purpose of this study was to provide a comprehensive overview of the safety of misoprostol. Adverse events related to misoprostol were collected from the FDA Adverse Event Reporting System (FAERS) database from the first quarter of 2004 to the second quarter of 2024. This study used proportional disequilibrium methods such as reporting odds ratio (ROR), proportional reporting ratio (PRR), Bayesian confidence propagation neural network (BCPNN), and empirical Bayes geometric mean (EBGM) to detect AEs. After analyzing 17,427,762 adverse event reports, a total of 2032 adverse events reports related to misoprostol were identified, involving 23 system organ classes and 30 preferred terms. The most common AEs were foetal exposure during delivery(*n* = 201), uterine tachysystole(*n* = 95), uterine rupture (*n* = 95), and heart rate decreased (*n* = 93). Although most AEs complied with the drug instruction, new AEs signals such as congenital aqueductal stenosis and congenital brain damage were also identified. Clinicians should make appropriate evaluation when using misoprostol, closely monitor the indicators of patients, and have appropriate countermeasures for possible adverse events.

**Supplementary Information:**

The online version contains supplementary material available at 10.1038/s41598-025-86422-z.

## Introduction

Misoprostol can inhibit gastric acid secretion. It was first marketed in the 1980s to prevent gastric ulcers^1^. Misoprostol, as a synthetic prostaglandin E1 analogue, is commonly used in obstetrical and gynecological diseases and has been widely used in abortion, cervical maturation, induced labour and postpartum hemorrhage^2^. The administration routes include oral, vaginal, rectal, or sublingual^2,3^. Vaginal misoprostol was a more effective option for cervical maturation and induced labor, but it had more maternal and neonatal complications^4^. Oral misoprostol caused fewer tachysystole than vaginal misoprostol and might reduce the occurrence of cesarean Sections^5^. Sublingual administration of misoprostol shortened delivery time without increasing related complications, and appeared to be superior to oral and vaginal routes^6^.

Misoprostol could cause uterine smooth muscle fibers to contract and cervical relaxation^7^. In terminating pregnancy, mifepristone was often combined with misoprostol^1^. The study showed that terminating pregnancy at home using misoprostol alone was also effective, safe and acceptable^8,9^. Even if the pregnancy was extended, the success rate was more than 95%^9^. In terms of fertility after abortion, misoprostol treatment was comparable to expected treatment^10^. Oral misoprostol was a safe choice for term prelabor rupture of the membranes (TPROM) induction, and the occurrence of adverse reactions could be reduced by adjusting the dose and frequency of administration^11^. Due to its low price, room temperature storage, and long shelf life, misoprostol was widely available worldwide^12,13^. Moreover, misoprostol could also be used to treat asthma and protect heart tissue from damage caused by paclitaxel and doxorubicin^14–16^.

The common adverse reactions of misoprostol included nausea, vomiting, diarrhea, headache, dizziness, etc.^17^. Some studies showed that misoprostol could cause cervical laceration, tachysystole, and uterine rupture in pregnant women^18–21^. In addition, the teratogenic risks of misoprostol also need to be taken seriously. Many studies showed that it could lead to fetal complications such as hydrocephalus, Moebius Syndrome, Holoprosencephaly, cleft lip and paddle, and brainstorm ischaemia, etc.^22–25^. However, the number of studies on misoprostol is limited, and there is currently a lack of comprehensive research on adverse reactions related to misoprostol.

Therefore, a comprehensive evaluation of the safety of misoprostol is necessary. By analyzing the FAERS data, adverse events (AEs) related to misoprostol were obtained. Doctors can understand the safety issues of misoprostol based on this. When using this drug in clinical practice, they can weigh the pros and cons, determine a better treatment plan, and seek greater benefits for patients.

## Materials and methods

### Data source

The FDA Adverse Event Reporting System (FAERS) is a database that includes reports of adverse drug events, medication errors, and more, updated quarterly^26^. The FAERS database receives voluntary reports from healthcare professionals, consumers and manufacturers^26^. We searched for American Standard Code for Information Interchange (ASCII) report files from the first quarter of 2004 to the second quarter of 2024, taking into account the time of market launch of misoprostol and the time of public data release from the FAERS database. Each ASCII file contains seven aspects of content, including Patient Demographic and Administrative Info (DEMO), DRUG (Drug/Biologic Info), MedDRA Terms for Adverse Event (REAC), Patient Outcomes (OUTC), Report Sources (RPSR), Drug Therapy Start/End Dates (THER), and MedDRA Terms for Diagnoses/Indications (INDI).

### Data extraction and analysis

We used the Medex_UIMA_1.3.8 system to standardize and unify drug names in the database. Data extraction was performed according to the drug name “misoprostol” and the drug must be the primary suspected (PS) drug. Deleting unrelated drugs or combination drugs, by specific keywords as follows: diclofe, naprosyn, mifepristone, eczema, naproxen, we obtained all drug information related to misoprostol. In addition, age groups were also divided as follows:<18, 18 ~ 55, 55 ~ 65,>=65. The measurement dates (days) for the occurrence time of adverse events were divided into the following: <7, 7 ~ 28, 28 ~ 60, >=60. Countries with a report count of ≥ 50 were presented. Reports of indications ≥ 30 were presented. In this study, the preferred term (PT) and system organ class (SOC) from the medical dictionary for regulatory activities (MedDRA26.1) were used to classify and describe signals of adverse events (AEs). We downloaded files from the FAERS database and removed duplicate records. For data with the same caseid in the DEMO table, only the most recent report based on the date was retained^27^. A single adverse event report may correspond to multiple adverse events. Some sum may not egaul the number of reports as they may be several items for one report as for exemple for outcomes.

Disproportionality analysis is a commonly used method for detecting drug warning risk signals in the FAERS database. The principle of the disproportionality method is to compare the degree of imbalance between the target drug and adverse events and other drugs and adverse events, in order to evaluate the correlation between the target drug and adverse events^28^. According to different statistical principles, it can be divided into the frequency method and the Bayesian method^28^. Both methods are based on the four grid table (Supplementary table S1) to quantify the degree of imbalance between the target drug and adverse events, in order to identify potential pharmacovigilance risk signals^28^. Both methods have their own advantages and disadvantages, and there is currently no gold standard for them. The frequency method has high sensitivity but low specificity, and is easily affected by individual values^28^. The Bayesian method has high specificity and stable signals, but its sensitivity is average^28^. This study used the reporting odds ratio (ROR) and proportional reporting ratio (PRR) in the frequency method, as well as the Bayesian confidence propagation neural network (BCPNN) and empirical Bayes geometric mean (EBGM) methods in the Bayesian method to detect the signals of AEs. The formulas and thresholds of the four algorithms are shown in Supplementary table S2. When four algorithms simultaneously detect a signal, it is the signal determined in this study. The joint use of multiple algorithms allows for cross-validation to reduce false positives^27^. A higher value indicates a stronger signal strength, suggesting a stronger association between the target drug and adverse events^27^. All analyses were performed using R 4.3.1.

## Results

### Characteristics on adverse event reports related to misoprostol

**Fig. 1:**
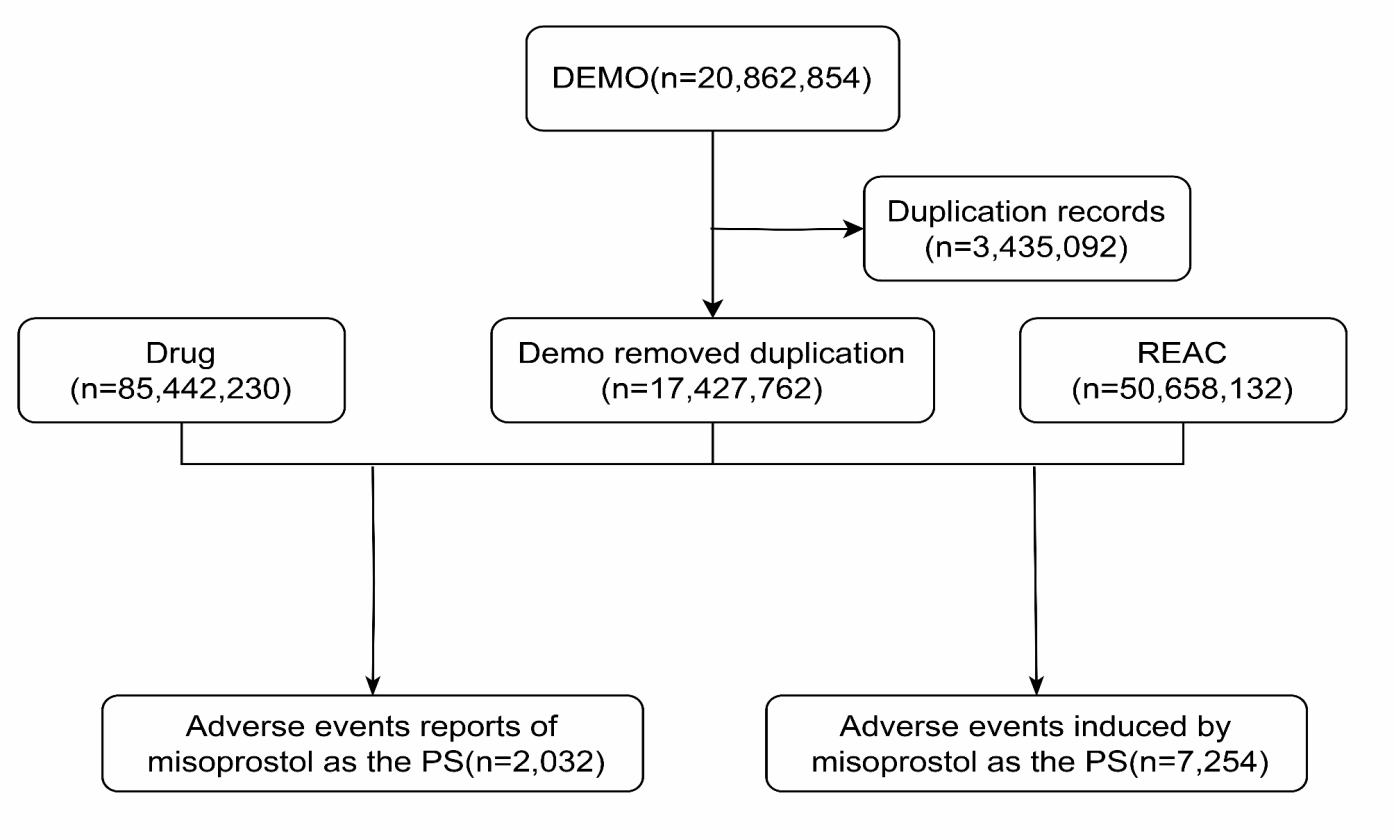
The flow diagram of selecting misoprostol-related AEs from the FAERS database; DEMO, Patient Demographic and Administrative Info; REAC, MedDRA Terms for Adverse Event.

We downloaded files from the first quarter of 2004 to the second quarter of 2024 in the FAERS database and deleted 3,435,092 duplicate records. For data with the same caseid in the DEMO table, only the most recent report based on the date was retained. This study obtained 17,427,762 adverse event reports from the FAERS database (Fig. 1). Among them, there were 2032 adverse events reports with misoprostol as the primary suspected (PS) drug (Fig. 1). The number of adverse events increased in recent years, peaking in 2020 (22.29%). From the perspective of gender distribution, females were the main group, accounting for 75.84%, while males accounted for 8.66%. In terms of age distribution, the incidence of adverse events was higher in the 18–55 age group (50.98%). Most of the reports were from consumers (38.98%), pharmacists (20.57%), and physicians (19.88%). The countries with the reports included Other (35.97%), Germany (22.29%), United States (18.65%), France (8.61%), China (4.87%), United Kingdom (3.69%), Italy (3.2%), and Denmark (2.71%) (top 8).

**Fig. 2:**
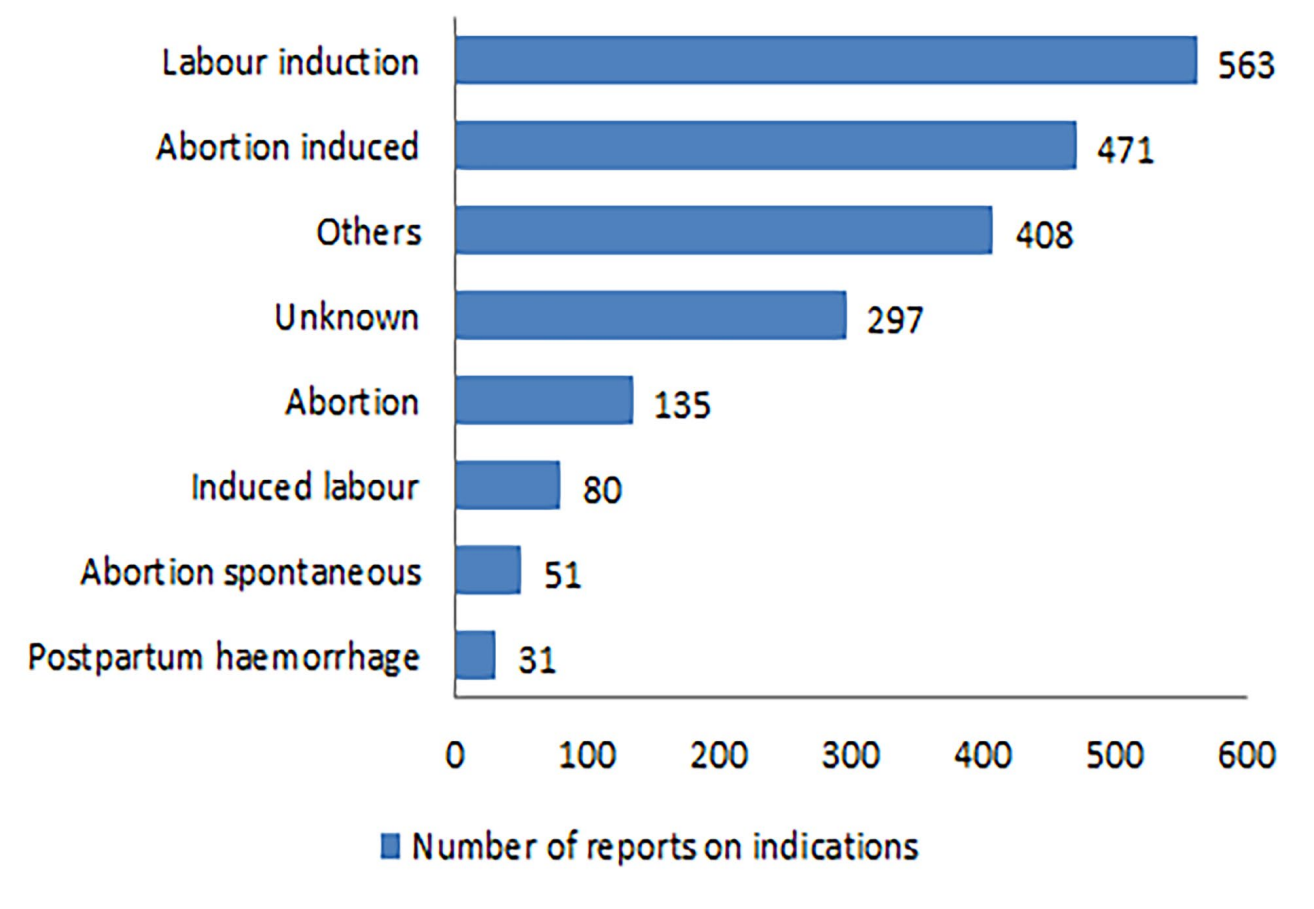
The indications related to misoprostol.

**Fig. 3:**
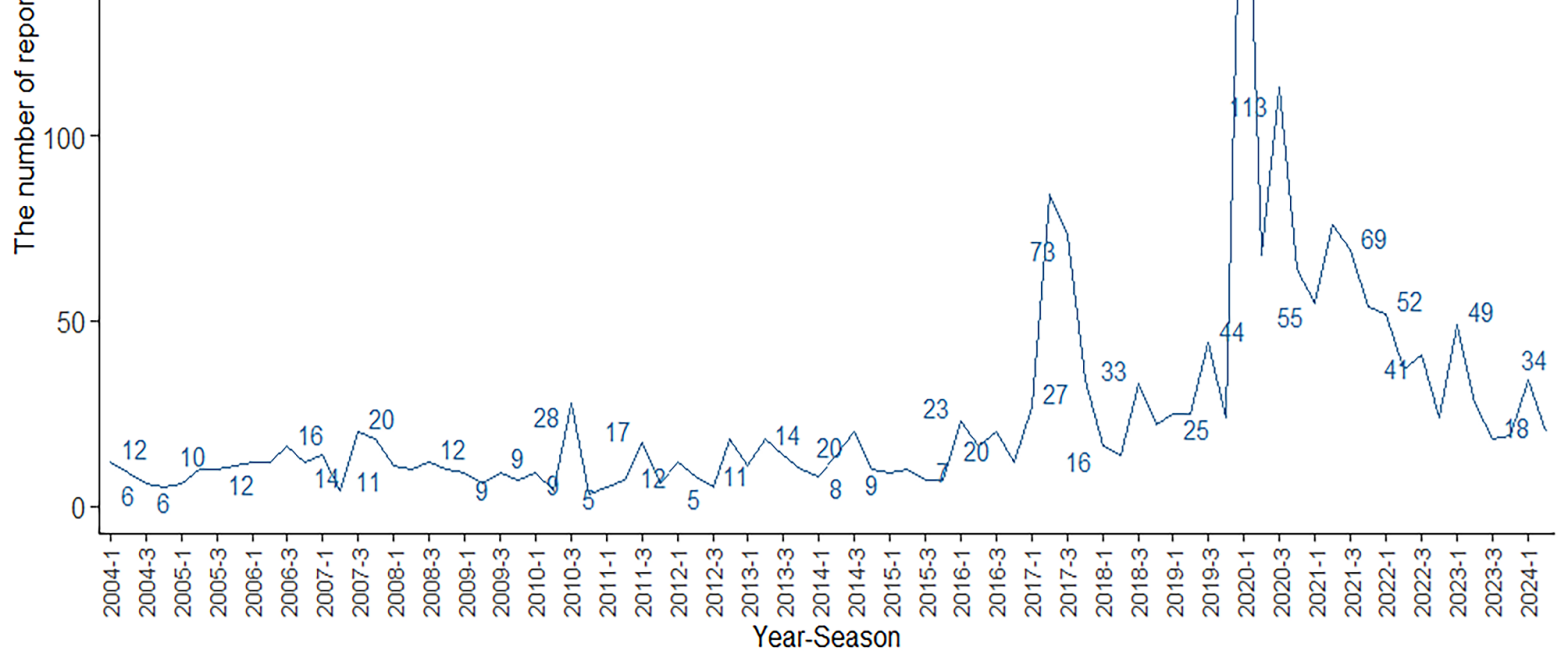
The number of adverse event reports for misoprostol per quarter.

In terms of route of administration, oral (26.33%), transplacental (20.08%), and vaginal (16.88%) were common. As for serious outcomes, such as other serious (53.67%) and hospitalization (23.78%) were common. AEs occurred mainly within 7 days after medication (65.25%). The indications of misoprostol were mainly related to labour induction (27.65%) and abortion induced (23.13%). Please refer to Table 1; Figs. 1 and 2 for specific information. The number of adverse event reports for misoprostol per quarter was shown in Fig. 3.

**Table 1:** Characteristics on adverse event reports related to misoprostol from the FAERS database.

Characteristics	Case number, *n*	Case proportion, %
Year		
2004	32	1.57
2005	37	1.82
2006	52	2.56
2007	56	2.76
2008	43	2.12
2009	31	1.53
2010	44	2.17
2011	35	1.72
2012	43	2.12
2013	53	2.61
2014	52	2.56
2015	33	1.62
2016	71	3.49
2017	218	10.73
2018	85	4.18
2019	118	5.81
2020	453	22.29
2021	254	12.5
2022	154	7.58
2023	114	5.61
2024	54	2.66
Gender		
Female	1541	75.84
Male	176	8.66
Unknown	315	15.5
Age		
<18	75	3.69
18 ~ 55	1036	50.98
55 ~ 65	37	1.82
>=65	47	2.31
Unknown	837	41.19
Reporter		
Consumer	792	38.98
Pharmacist	418	20.57
Physician	404	19.88
Other health-professional	321	15.8
Unknown	72	3.54
Lawyer	24	1.18
Registered Nurse	1	0.05
Reported countries		
Other	731	35.97
Germany	453	22.29
United States	379	18.65
France	175	8.61
China	99	4.87
United Kingdom	75	3.69
Italy	65	3.20
Denmark	55	2.71
Route		
Other	639	31.45
Oral	535	26.33
Transplacental	408	20.08
Vaginal	343	16.88
Sublingual	50	2.46
Buccal	35	1.72
Rectal	22	1.08
Outcomes		
Other serious	1264	53.67
Hospitalization	560	23.78
Life threatening	187	7.94
Death	143	6.07
Congenital anomaly	94	3.99
Disability	86	3.65
Required intervention to Prevent Permanent Impairment/Damage	21	0.89
Adverse event occurrence time - medication date (days)		
<7	813	65.25
7 ~ 28	32	2.57
28 ~ 60	13	1.04
>=60	22	1.77
Unknown	366	29.37
Indications		
Abortion	135	6.63
Abortion induced	471	23.13
Abortion spontaneous	51	2.50
Induced labour	80	3.93
Labour induction	563	27.65
Others	408	20.04
Postpartum haemorrhage	31	1.52
Unknown	297	14.59

### Signals detection

#### Signals detection at SOC levels

This study found that AEs associated with misoprostol mainly involved 23 system organ classes (SOCs) (Table 2). Among them, there were four systems that showed strong correlation with the four signal recognition methods, as follows: pregnancy, puerperium and perinatal conditions (*n* = 759, ROR 25.81, PRR 23.21, IC 4.53, EBGM 23.14), congenital, familial and genetic disorders (*n* = 150, ROR 6.51, PRR 6.4, IC 2.68, EBGM 6.39), injury, poisoning and procedural complications (*n* = 2114, ROR 3.87, PRR 3.03, IC 1.6, EBGM 3.03), and reproductive system and breast disorders (*n* = 236, ROR 3.85, PRR 3.76, IC 1.91, EBGM 3.76) (Table 2).

**Table 2:** The signal strength of AEs of misoprostol at the SOC level.

System organ class	Case reports	ROR (95% CI)	PRR (95% CI)	χ^2^	IC (IC025)	EBGM (EBGM05)
Pregnancy, puerperium and perinatal conditions	759	25.81(23.93, 27.82)	23.21(21.88, 24.62)	16151.37	4.53(4.42)	23.14(21.72)
Congenital, familial and genetic disorders	150	6.51(5.54, 7.66)	6.4(5.47, 7.49)	684.75	2.68(2.44)	6.39(5.58)
Injury, poisoning and procedural complications	2114	3.87(3.68, 4.07)	3.03(2.91, 3.15)	3183.34	1.6(1.53)	3.03(2.91)
Reproductive system and breast disorders	236	3.85(3.39, 4.39)	3.76(3.34, 4.23)	482.35	1.91(1.72)	3.76(3.37)
Vascular disorders	283	1.75(1.56, 1.97)	1.72(1.53, 1.93)	87.96	0.79(0.61)	1.72(1.56)
Cardiac disorders	275	1.37(1.21, 1.54)	1.35(1.2, 1.52)	26.19	0.44(0.26)	1.35(1.22)
Respiratory, thoracic and mediastinal disorders	316	0.87(0.78, 0.97)	0.87(0.77, 0.98)	6	−0.19(−0.36)	0.87(0.8)
Investigations	399	0.84(0.76, 0.93)	0.85(0.77, 0.94)	11.61	−0.24(−0.38)	0.85(0.78)
Nervous system disorders	512	0.78(0.71, 0.85)	0.79(0.73, 0.85)	29.84	−0.33(−0.46)	0.79(0.74)
Immune system disorders	64	0.76(0.59, 0.97)	0.76(0.6, 0.96)	4.77	−0.39(−0.74)	0.76(0.62)
Gastrointestinal disorders	436	0.65(0.59, 0.72)	0.67(0.61, 0.74)	75.93	−0.57(−0.71)	0.67(0.62)
General disorders and administration site conditions	814	0.57(0.53, 0.61)	0.62(0.58, 0.66)	235.16	−0.69(−0.8)	0.62(0.58)
Infections and infestations	183	0.45(0.39, 0.52)	0.46(0.4, 0.53)	122.83	−1.12(−1.33)	0.46(0.41)
Blood and lymphatic system disorders	57	0.44(0.34, 0.57)	0.44(0.34, 0.57)	40.68	−1.18(−1.55)	0.44(0.36)
Ear and labyrinth disorders	14	0.43(0.25, 0.72)	0.43(0.25, 0.73)	10.75	−1.22(−1.95)	0.43(0.28)
Endocrine disorders	8	0.42(0.21, 0.83)	0.42(0.21, 0.83)	6.52	−1.26(−2.2)	0.42(0.23)
Skin and subcutaneous tissue disorders	160	0.38(0.33, 0.44)	0.39(0.33, 0.46)	158	−1.34(−1.57)	0.39(0.35)
Psychiatric disorders	165	0.37(0.31, 0.43)	0.38(0.32, 0.44)	177.57	−1.4(−1.62)	0.38(0.33)
Musculoskeletal and connective tissue disorders	151	0.36(0.31, 0.43)	0.38(0.32, 0.44)	164.71	−1.41(−1.64)	0.38(0.33)
Renal and urinary disorders	42	0.3(0.22, 0.4)	0.3(0.22, 0.4)	69.89	−1.74(−2.17)	0.3(0.23)
Metabolism and nutrition disorders	46	0.28(0.21, 0.37)	0.28(0.21, 0.38)	85.7	−1.82(−2.24)	0.28(0.22)
Hepatobiliary disorders	18	0.26(0.16, 0.41)	0.26(0.16, 0.42)	38.29	−1.94(−2.59)	0.26(0.18)
Eye disorders	37	0.24(0.17, 0.33)	0.24(0.18, 0.33)	88.08	−2.03(−2.49)	0.24(0.19)
Neoplasms benign, malignant and unspecified (incl cysts and polyps)	15	0.07(0.04, 0.12)	0.07(0.04, 0.12)	178.51	−3.75(−4.46)	0.07(0.05)

### Signals detection at PTs level

The study ranked the top 30 PTs in descending order based on ROR (Table 3). The results showed that uterine tachysystole(*n* = 95, ROR 19203.92, PRR 18952.43, IC 12.32, EBGM 5103.31), abortion induced incomplete (*n* = 13, ROR 4546.76, PRR 4538.61, IC 11.43, EBGM 2751.07), foetal heart rate decreased(*n* = 93, ROR 1334.27, PRR 1317.18, IC 10.11, EBGM 1108.3), endometritis bacterial (*n* = 3, ROR 3492.68, PRR 3491.24, IC 11.18, EBGM 2327.83), and postpartum stress disorder (*n* = 9, ROR 6991.15, PRR 6982.48, IC 11.77, EBGM 3491.74) were high signals (Table 3). The most common AEs were foetal exposure during delivery(*n* = 201), uterine tachysystole(*n* = 95), uterine rupture (*n* = 95), and heart rate decreased(*n* = 93) (Table 3). In addition to the adverse reactions mentioned in the instructions, this study also discovered various congenital, familial and genetic disorders, such as congenital aqueductal stenosis and congenital brain damage (Table 3).

**Table 3:** The top 30 signal strength of AEs of misoprostol ranked by ROR at the PTs level.

System organ class	PTs	Case reports	ROR (95% CI)	PRR (95% CI)	χ^2^	IC (IC025)	EBGM (EBGM05)
Pregnancy, puerperium and perinatal conditions	Uterine tachysystole	95	19203.92 (13024.98, 28314.1)	18952.43 (12806.23, 28048.42)	484695.03	12.32 (11.94)	5103.31 (3687.8)
Pregnancy, puerperium and perinatal conditions	Birth trauma	16	2332.63 (1324.09, 4109.37)	2327.49 (1318.36, 4109.04)	27905.94	10.77 (10)	1745.87 (1087.01)
Pregnancy, puerperium and perinatal conditions	Traumatic delivery	10	2330.7 (1138.95, 4769.45)	2327.49 (1149.34, 4713.31)	17441.21	10.77 (9.81)	1745.87 (958.96)
Pregnancy, puerperium and perinatal conditions	Precipitate labour	15	2141.92 (1200.67, 3821.06)	2137.49 (1210.74, 3773.61)	24524.83	10.68 (9.89)	1636.75 (1008.43)
Pregnancy, puerperium and perinatal conditions	Post abortion haemorrhage	29	1848.22 (1226.83, 2784.33)	1840.83 (1219.71, 2778.24)	42200.72	10.51 (9.94)	1456.98 (1034.05)
Pregnancy, puerperium and perinatal conditions	Failed trial of labour	3	1746.34 (492.7, 6189.82)	1745.62 (488.27, 6240.81)	4184.69	10.45 (8.87)	1396.7 (484.48)
Pregnancy, puerperium and perinatal conditions	Uterine hyperstimulation	16	1492.89 (869.77, 2562.4)	1489.59 (860.45, 2578.74)	19616.68	10.26 (9.51)	1227.86 (781.33)
Pregnancy, puerperium and perinatal conditions	Abnormal labour	19	1278.99 (783.9, 2086.78)	1275.64 (781.49, 2082.25)	20461.21	10.08 (9.39)	1078.75 (716.18)
Pregnancy, puerperium and perinatal conditions	Labour pain	10	932.28 (481.74, 1804.2)	931 (478.12, 1812.86)	8197.05	9.68 (8.78)	821.59 (472.86)
Pregnancy, puerperium and perinatal conditions	Anaphylactoid syndrome of pregnancy	10	908.07 (469.7, 1755.56)	906.82 (465.7, 1765.77)	8008.16	9.65 (8.74)	802.7 (462.38)
Pregnancy, puerperium and perinatal conditions	Uterine atony	20	660.54 (417.38, 1045.36)	658.72 (419.68, 1033.91)	12002.28	9.23 (8.59)	602.02 (410.01)
Pregnancy, puerperium and perinatal conditions	Uterine hypertonus	19	556.55 (348.63, 888.48)	555.09 (346.79, 888.5)	9734.93	9.01 (8.35)	514.29 (347.72)
Injury, poisoning and procedural complications	Abortion induced incomplete	13	4546.76 (2260.89, 9143.73)	4538.61 (2241.22, 9190.96)	35743.01	11.43 (10.53)	2751.07 (1533.27)
Injury, poisoning and procedural complications	Induced abortion haemorrhage	8	3994.39 (1675.16, 9524.56)	3989.99 (1684.37, 9451.64)	20302.48	11.31 (10.2)	2539.45 (1227.33)
Injury, poisoning and procedural complications	Foetal exposure during delivery	201	3749.26 (3156.32, 4453.57)	3645.4 (3055.88, 4348.65)	481139.09	11.23 (10.99)	2395.36 (2074.02)
Injury, poisoning and procedural complications	Induced abortion failed	43	3595.67 (2488.01, 5196.46)	3574.36 (2463.01, 5187.16)	101601.63	11.21 (10.71)	2364.48 (1737.48)
Injury, poisoning and procedural complications	Uterine cervical laceration	10	1109.86 (569.3, 2163.69)	1108.33 (569.19, 2158.16)	9547.8	9.9 (8.99)	956.64 (547.21)
Injury, poisoning and procedural complications	Perineal injury	15	971.8 (565.99, 1668.55)	969.79 (560.19, 1678.88)	12746.52	9.73 (8.98)	851.64 (541.78)
Injury, poisoning and procedural complications	Uterine rupture	95	525.09 (425.76, 647.61)	518.23 (417.72, 642.92)	45654.97	8.91 (8.61)	482.49 (404.84)
Investigations	Foetal heart rate decreased	93	1334.27 (1067.79, 1667.26)	1317.18 (1061.72, 1634.1)	102901.82	10.11 (9.8)	1108.3 (919.81)
Investigations	Amniocentesis abnormal	15	783.24 (459.11, 1336.19)	781.62 (460.43, 1326.86)	10517.08	9.46 (8.71)	703.03 (449.65)
Investigations	Foetal heart rate increased	6	551.7 (240.22, 1267.09)	551.25 (242.01, 1255.62)	3054.37	9 (7.88)	510.99 (254.84)
Infections and infestations	Endometritis bacterial	3	3492.68 (873.32, 13968.3)	3491.24 (868.19, 14039.25)	6978.48	11.18 (9.51)	2327.83 (729.88)
Infections and infestations	Post abortion infection	7	2877.91 (1193.09, 6941.93)	2875.14 (1190.18, 6945.55)	14245.98	10.99 (9.84)	2036.85 (974.96)
Infections and infestations	Endometritis	39	501.44 (362.06, 694.48)	498.75 (357.42, 695.97)	18081.92	8.86 (8.4)	465.57 (354.51)
Congenital, familial and genetic disorders	Congenital aqueductal stenosis	3	1309.76 (381.55, 4496.03)	1309.21 (380.84, 4500.66)	3302.44	10.11 (8.56)	1102.65 (392.87)
Congenital, familial and genetic disorders	Congenital brain damage	7	514.99 (238.94, 1110)	514.5 (239.56, 1104.99)	3341.31	8.9 (7.87)	479.26 (252.06)
Psychiatric disorders	Postpartum stress disorder	9	6991.15 (2774.32, 17617.36)	6982.48 (2779.31, 17542.11)	31412.15	11.77 (10.66)	3491.74 (1611.32)
Endocrine disorders	Postpartum hypopituitarism	3	1047.8 (311.29, 3526.93)	1047.37 (310.7, 3530.65)	2727.06	9.83 (8.3)	910.89 (329.92)
Cardiac disorders	Foetal heart rate disorder	11	545.56 (295.24, 1008.1)	544.73 (296.69, 1000.13)	5538.05	8.98 (8.13)	505.38 (302.34)

## Discussion

Misoprostol was originally used to treat gastric ulcers, and had been widely used in abortion, cervical maturation, induced labour and postpartum hemorrhage^1,2^. In addition to the above indications, misoprostol also played a role in other areas. A study showed that the high-dose of misoprostol could relieve asthma by reducing IL-5 levels^14^. Animal experiments showed that misoprostol had antioxidant and anti-apoptotic effects and could protect rat heart from paclitaxel and doxorubicin damage^15,16^.

With more and more widely used, its adverse reactions have gradually attracted people’s attention. In addition to the common gastrointestinal adverse reactions, other adverse reactions such as skin reactions, tachysystole, uterine rupture, teratogenesis and so on were also increasing. Some pregnant women experienced erythroma multiforme and toxic epidermal necrolysis after taking misoprostol orally during termination of pregnancy^29,30^. 140 cases (31.4%) of women experienced tachystole after induction of labor using misoprostol^20^. A meta-analysis suggested that vaginal misoprostol led to a higher incidence of tachysystole [OR: 1.48;95% CI: 1.09–2.01]^31^. A 49 year old woman experienced sudden hypotension and subsequently developed anaphylactic shock while using misoprostol to promote cervical ripening before hysteroscopy^32^. Misoprostol could cause uterine rupture in primipara^33^. Some pregnant women continued pregnancy after oral misoprostol, exposing the fetus to misoprostol, which might lead to congenital abnormalities, such as: Hydrocephalus, Moebius Syndrome and Holoprosencephaly, neural tube defects, heart malformation, and cleft lip and palate, etc.^22–24,34^. A study in Brazil showed a positive correlation between misoprostol and congenital abnormalities, with fetuses exposed to misoprostol having a 2.74 times higher risk of developing congenital abnormalities compared to those not exposed^35^. However, another study in Brazil revealed that there was no evidence of a strong teratogenic effect of misoprostol exposure during pregnancy, and the risk of congenital abnormalities increased by misoprostol was very low^36^. This study systematically evaluated adverse events related to misoprostol through in-depth analysis of the FAERS database from the first quarter of 2004 to the second quarter of 2024. It provided more accurate data support for clinical practice and public health decision-making.

- This study found that misoprostol related adverse events increased significantly in recent years and reached a peak in 2020, which might be due to the fact that patients were mainly at home during the COVID-19 period and their activity space was restricted. A study only using misoprostol for medical abortion from December 2020 to December 2021 showed that among the 911 patients included, up to 90% had complete abortion, and three patients experienced adverse events requiring blood transfusion^37^. A network pharmacology study revealed that the core therapeutic targets of misoprostol for terminating pregnancy were HSP90AA1, EGFR, and MAPK1, which interfered with protein phosphorylation, cell localization, and protein hydrolysis regulation processes through the VEGF signaling pathway, calcium signaling pathway, and NF-κB signaling pathway^38^.
- Misoprostol related AEs were more common in female patients because this drug was currently widely used to treat gynecological and obstetric diseases. Misoprostol was used in patients aged 18–55 years and was mostly used in adult women of reproductive age, which was in line with its current widely used indications. Most of the reports were from consumers, indicating that patients were more inclined to report adverse reactions directly after using misoprostol and had a higher level of awareness. Except other countries, the countries with more reports were Germany and the United States, which indicated that economically developed countries might pay more attention to adverse drug reactions, which was also conducive to other countries to strengthen their attention to adverse drug reactions. The main routes of administration for misoprostol were oral, transplacetal, and vaginal. Oral misoprostol induced labor was superior to vaginal administration in neonatal death, tachysystole, and preeclampsia, and had fewer adverse reactions in pregnant women and newborns^39^. The main indications were Labour induction and Abortion induced, which were consistent with the clinical indications of misoprostol. The common outcomes of taking misoprostol were hospitalization (23.78%), life threatening (7.94%), and death (6.07%), indicating that the adverse reactions of misoprostol should be taken seriously by both doctors and patients.

Misoprostol, as a commonly used drug in obstetrics and gynecology, had AEs mainly concentrated in pregnancy, puerperium and peripheral conditions, congenital, familial and genetic disorders, injury, poisoning and procedural complexes, and reproductive system and breast disorders, among other SOCs, and most of them were included in the drug instructions, indicating the reliability of the results of this study. At the PTs level, The most common AEs were foetal exposure during delivery (*n* = 201), uterine tachysystole (*n* = 95), uterine rupture (*n* = 95) and foetal heart rate decreased (*n*= 93), partially consistent with the drug instructions and previous studies. After using misoprostol for cervical maturation, 32% of patients experienced fetal heart tracin abnormalities, and three cases of tachystole and two cases of pelvic abruption occurred^40^. Oral or vaginal misoprostol could cause uterine rupture during induced abortion, and women with a history of cesarean section were more likely to experience uterine rupture^41–43^. About 15% of misoprostol induced abortions might fail, leading to fetal exposure to the drug and potentially inducing birth defects^44^. A meta-analysis showed that the use of misoprostol increased the risk of congenital malformations (OR = 3.56; 95% CI: 0.98–12.98), and prenatal exposure to misoprostol was associated with an increased risk of Mobius sequence and terminal transverse limb defects^45^. A study suggested that congenital malformations in Brazilian children were associated with abuse of misoprostol in early pregnancy^25^.

In addition, this study also found adverse events that were not documented in the drug instructions, such as postpartum hypopituitarism, postpartum stress disorder, amniocentesis abnormal, endometritis bacterial, and anaphylactoid syndrome of pregnancy. No literature reports have been found on misoprostol and postpartum hypopituitarism, postpartum stress disorder, and amniocentesis abnormal. Medical abortion could lead to endometritis. Two young women underwent a combination of mifepristone and misoprostol medical abortion in early pregnancy^46,47^. After the abortion, they showed symptoms of infection^46,47^. Gram positive cocci and pyogenic streptococcus (Group A streptococcus) were cultured in the uterine trophoblast tissue, which might be caused by endouterine migration from a preexisting colonization of the vaginal flora or exogenous contamination during the procedure^46,47^. Relevant prevention and hygiene measures were crucial^46,47^. Four deaths associated with C. sordellii endometritis and toxic shock syndrome occurred within one week after medically induced abortions^48^. Clinically, doctors should enhance their understanding of this clinical manifestation and conduct in-depth research on its relationship with medical abortion^48^. Anaphylactoid syndrome of pregnancy was previously referred to as amniotic fluid embolism. A 27 year old primiparous woman developed amniotic fluid embolism after vaginal misoprostol to enhance uterine contractions, but unfortunately passed away despite all efforts to save her^49^. Another 26 year old primiparous woman experienced amniotic fluid embolism after vaginal misoprostol to promote cervical maturation and induce uterine contractions^50^. After rescue, the patient turned danger into safety^50^. These suggested that clinical training for medical staff should be strengthened to timely detect early symptoms of amniotic fluid embolism, treat them as soon as possible, and reduce maternal mortality^49^. In addition, the hospital’s obstetrics ward, blood bank, and operating room should meet the needs of rescuing patients^49^. These AEs all showed high signals, revealing their potential risk information. Adverse events that were not recorded in the instructions but were considered high signal in this study should be taken seriously by clinical doctors to reduce potential adverse outcomes in clinical practice.

This study provided strong support for the safety of misoprostol by analyzing real-world data in detail through the FAERS database. However, this study still had some limitations. Firstly, spontaneous reporting could lead to data bias, and the data provided by healthcare professionals might be more comprehensive and reliable than data from other sources. Secondly, the submitted report might not be detailed enough and might not accurately assess adverse events. It was also impossible to calculate the incidence of adverse events. Besides, the Medicines and Healthcare Products Regulatory Agency released a Drug Safety Update on Misoprostol on 6 February 2018, as follows: monitor patients closely and remove the vaginal delivery system immediately in cases of excessive or prolonged uterine contractions at the onset of labour, or if there is clinical concern for mother or baby^51^. The safety alerts of misoprostol might affect its use in obstetrics. Also, this study did not conduct sensitivity analysis on pooled indications at HLT levels of MedDRA. The lack of sensitivity analysis with a reference group containing only drugs with the same indicators was also the limitation of this study. In addition, multiple testing could accumulate the possibility of false positives, leading to an overall increase in error rates. Furthermore, we were unable to conduct a case review of reports and verify the signals. Finally, the causal relationship between misoprostol and adverse events could not be determined, and further studies were needed to explore the causal relationship. Despite these limitations, the results of this study had certain reference value for the clinical use of misoprostol by medical personnel.

## Conclusion

This study analyzed the AEs data related to misoprostol in the FAERS database and found that most of the SOCs involved were included in the drug instructions, indicating the reliability of the results of this study. This study also found adverse events that were not documented in the drug instructions, such as postpartum hypopituitarism, postpartum stress disorder, amniocentesis abnormal, anaphylactoid syndrome of pregnancy, and endometritis bacterial. These AEs all showed high signals, revealing their potential risk information. Clinicians should make appropriate evaluation when using misoprostol, closely monitor the indicators of patients, and have appropriate countermeasures for possible adverse events.

## Electronic supplementary material

Below is the link to the electronic supplementary material.

Supplementary Material 1

Supplementary Material 2

## Data Availability

The study used data from the FDA Adverse Event Reporting System (FAERS) database. The raw data supporting the conclusions of this article will be made available by the corresponding author, without undue reservation.
