# Supplementary material for: A disproportionality analysis of FDA adverse event reporting system events for misoprostol
Source: Sci Rep. 2025 Jan 19;15:2452. doi: 10.1038/s41598-025-86422-z (PMC11743753; doi:10.1038/s41598-025-86422-z)
Supplement: Supplementary file 1 — Supplementary Material 1 [file 41598_2025_86422_MOESM1_ESM.pdf]

Supplementary table S1. The four grid table.

	Adverse event reports for the target	Other adverse event reports	Total
Misoprostol	a	b	a + b
Other drugs in the FAERS database	c	d	c + d
Total	a + c	b + d	N = a + b + c + d

Notes:

a. Number of adverse reaction reports for the target drug;

b. Number of other adverse reaction reports for the target drug;

c. Number of adverse reaction reports for non target drugs;

d. Number of other adverse reaction reports for non target drugs.
