# Supplementary material for: A disproportionality analysis of FDA adverse event reporting system events for misoprostol
Source: Sci Rep. 2025 Jan 19;15:2452. doi: 10.1038/s41598-025-86422-z (PMC11743753; doi:10.1038/s41598-025-86422-z)
Supplement: Supplementary file 2 — Supplementary Material 2 [file 41598_2025_86422_MOESM2_ESM.pdf]

Supplementary table S2. The formulas and thresholds of the four algorithms.

Method	Formula	Threshold
ROR	$\mathrm{ROR} = \frac{\mathrm{a}/\mathrm{c}}{b/d}$	a ≥ 3<br>ROR ≥ 3<br>95%CI<br>(lower limit) > 1
	$SE(lnROR) = \sqrt{\frac{1}{\mathrm{a}} + \frac{1}{\mathrm{b}} + \frac{1}{\mathrm{c}} + \frac{1}{\mathrm{d}}}$	
	$95\%CI = \mathrm{e}^{\ln(ROR) \pm 1.96se}$	
PRR	$\mathrm{PRR} = \frac{\mathrm{a}/(\mathrm{a}+\mathrm{b})}{c/(c+d)}$	a ≥ 3<br>PRR ≥ 2<br>95%CI<br>(lower limit) > 1
	$SE(lnPRR) = \sqrt{\frac{1}{\mathrm{a}} - \frac{1}{\mathrm{a}+\mathrm{b}} + \frac{1}{\mathrm{c}} - \frac{1}{\mathrm{c}+\mathrm{d}}}$	
	$95\%CI = \mathrm{e}^{\ln(PRR) \pm 1.96se}$	
BCPNN	$\mathrm{IC} = \log_2 \frac{p(x,y)}{p(x)p(y)} = log_2 \frac{a(a+b+c+d)}{(a+b)(a+c)}$	IC025>0
	$\mathrm{E(IC)} = \log_2 \frac{(a+\gamma 11)(a+b+c+d+\alpha)(a+b+c+d+\beta}{(a+b+c+d+\gamma)(a+b+\alpha 1)(a+c+\beta 1)}$	
	$\mathrm{V(IC)} = \frac{1}{(ln2)^2}[\frac{(a+b+c+d)-a+\gamma-\gamma 11}{(a+\gamma 11)(1+a+b+c+d+\gamma)} + \frac{(a+b+c+d)-(a+b)+a-\alpha 1}{(a+b+\alpha 1)(1+a+b+c+d+\alpha)} + \frac{(a+b+c+d+\alpha)-(a+c)+\beta-\beta 1}{(a+b+\beta 1)(1+a+b+c+d+\beta)}]$	
	$\gamma = \gamma 11 \frac{(a+b+c+d+\alpha)(a+b+c+d+\beta)}{(a+b+\alpha 1)(a+c+\beta 1)}$	
	$\mathrm{IC} - 2\mathrm{SD} = \mathrm{E(IC)} - 2\sqrt{\mathrm{V(IC)}}$	
EBGM	$\mathrm{EBGM} = \frac{a(a+b+c+d)}{(a+c)(a+b)}$	EBGM05>2
	$SE(lnEBGM) = \sqrt{\frac{1}{\mathrm{a}} + \frac{1}{\mathrm{b}} + \frac{1}{\mathrm{c}} + \frac{1}{\mathrm{d}}}$	
	$95\%CI = \mathrm{e}^{\ln(EBGM) \pm 1.96se}$	
